# Whole-genome sequencing of clinical isolates from tuberculosis patients in India: real-world data indicates a high proportion of pre-XDR cases

**DOI:** 10.1128/spectrum.02770-23

**Published:** 2024-04-10

**Authors:** Aparna Bhanushali, Sachin Atre, Preethi Nair, Geethanjali Anilkumar Thandaseery, Sanchi Shah, Sanjana Kuruwa, Amrutraj Zade, Chaitali Nikam, Mangala Gomare, Anirvan Chatterjee

**Affiliations:** 1 HaystackAnalytics Pvt. Ltd., IIT Bombay, Mumbai, India; 2 Dr. D.Y. Patil Medical College Hospital and Research Centre, Pune, India; 3 Thyrocare Technologies Ltd., Navi Mumbai, India; 4 BrihanMumbai Municipal Corporation, Mumbai, India; Petrified Bugs LLC, Miami, Florida, USA

**Keywords:** whole-genome sequencing, multidrug resistant, *Mycobacterium tuberculosis*, India, molecular biology, drug-resistance mechanisms, drug-sensitivity testing, molecular diagnostics, phylogeny

## Abstract

Treatment decisions for tuberculosis (TB) in the absence of full drug-susceptibility data can result in amplifying resistance and may compromise treatment outcomes. Genomics of *Mycobacterium tuberculosis* (*M.tb*) from clinical samples enables detection of drug resistance to multiple drugs. We performed whole-genome sequencing (WGS) for 600 clinical samples from patients with tuberculosis to identify the drug-resistance profile and mutation spectrum. We documented the reasons reported by clinicians for referral. WGS identified a high proportion (51%) of pre-extensively drug-resistant (pre-XDR) cases followed by multidrug-resistant tuberculosis (MDR-TB) (15.5%). This correlates with the primary reason for referral, as non-response to the first-line treatment (67%) and treatment failure or rifampicin resistance (14%). Multivariate analysis indicated that all young age groups (*P* < 0.05), male gender (*P* < 0.05), and Beijing strain (*P* < 0.01) were significant independent predictors of MDR-TB or MDR-TB+ [pre-extensively drug-resistant tuberculosis (XDR-TB) and XDR-TB]. Ser315Thr (72.5%) in the *inhA* gene and Ser450Leu in the *rpoB* gene (65.5%) were the most prevalent mutations, as were resistance-conferring mutations to pyrazinamide (41%) and streptomycin (61.33%). Mutations outside the rifampicin resistance-determining region (RRDR), Ile491Phe and Val170Phe, were seen in 1.3% of cases; disputed mutations in *rpoB* (Asp435Tyr, His445Asn, His445Leu, and Leu430Pro) were seen in 6% of cases, and mutations to newer drugs such as bedaquiline and linezolid in 1.0% and 7.5% of cases, respectively. This study on clinical samples highlights that there is a high proportion of pre-XDR cases and emerging resistance to newer drugs; ongoing transmission of these strains can cause serious threat to public health; and whole-genome sequencing can effectively identify and support precision medicine for TB.

**IMPORTANCE:**

The current study is based on real-world data on the TB drug-resistance profile by whole-genome sequencing of 600 clinical samples from patients with TB in India. This study indicates the clinicians’ reasons for sending samples for WGS, which is for difficult-to-treat cases and/or relapse and treatment failure. The study reports a significant proportion of cases with pre-XDR-TB strains that warrant policy makers’ attention. It reflects the current iterative nature of the diagnostic tests under programmatic conditions that leads to delays in appropriate diagnosis and empirical treatment. India had an estimated burden of 2.95 million TB cases in 2020 and 135,000 multidrug-resistant cases. However, WGS profiles of *M.tb* from India remains disproportionately poorly represented. This study adds a significant body of data on the mutation profiles seen in *M.tb* isolated from patients with TB in India, mutations outside the RRDR, disputed mutations, and resistance-conferring mutations to newer drugs such as bedaquiline and linezolid.

## INTRODUCTION

The emergence of drug-resistant tuberculosis (DR-TB) is a global health crisis, more so in India, which shares one-fourth of the global tuberculosis (TB) burden (27%), with an estimated 2.95 million TB cases in 2020 and an estimated 135,000 multidrug-resistant (MDR) cases (1). MDR-TB is defined as strains of *Mycobacterium tuberculosis* (*M.tb*) that are resistant to at least isoniazid (INH) and rifampicin (RMP), while extensively drug-resistant tuberculosis (XDR-TB) denotes strains with additional resistance to fluoroquinolones and at least one group A drug [(levofloxacin or moxifloxacin (MOX), bedaquiline (BDQ), and linezolid (LZD)].

Treatment of DR-TB cases is guided by the programmatic management of drug-resistant tuberculosis, in line with global recommendations of several regimens including newer drugs for DR-TB (2). With three new and recently repurposed drugs, treatment of MDR-TB is more hopeful today than at any time. Despite this, global treatment success rates have been reported only between 48% and 56% by the World Health Organization (WHO) in its annual Global Tuberculosis Reports from 2014 to 2019.

A previous study from India in the state of Maharashtra among 4,024 MDR-TB cases under the Revised National TB Control Program showed that the treatment success rate with 24-month standard regimen was even poorer (29%) than the global rates (3, 4). One of the many reasons for poor treatment outcome is poor diagnosis seen in a large number of MDR-TB cases (5)

Early and comprehensive universal drug-susceptibility testing is expected to significantly improve treatment outcome and reduce transmission of drug-resistant forms of TB (6). However, India faces considerable challenges because of low investment in public health programs, high endemicity in difficult-to-reach locations, and persistence of diagnostic gaps despite the National Tuberculosis Elimination Programme (NTEP) initiative for expansion of diagnostic network facilities to provide quality-assured culture and drug-susceptibility testing and rapid molecular assays. Although the rollout of these technologies has led to the early detection of a large number of MDR-TB/XDR-TB cases and their enrollment on treatment, an estimated 56% of MDR-TB cases in India remain undiagnosed (7).

Next-generation sequencing (NGS) can detect resistance to almost all the drugs simultaneously. Tuberculosis whole-genome sequencing (TB-WGS) has become the standard of care in several developed countries (8, 9). Since whole-genome sequencing (WGS) can interrogate the entire genome and identify drug resistance-conferring mutations, TB-WGS plays a crucial role in surveillance and personalized treatment (10).

The release of guidelines for genomic analysis of drug resistance in *M.tb* (8) and widely reported benefits of WGS suggest that there is a growing need of this technology among treating pulmonologists. Here we report the WGS of 600 *M*.*tb* isolates from such referral cases. We report the drug-resistance profile of these isolates and the significant association between patient types and strain types. We highlight the reasons and motivation of treating pulmonologists for such referrals which can help shape the health strategies and policies.

## MATERIALS AND METHODS

### Study setting

This study was conducted by HaystackAnalytics in collaboration with Dr. D.Y. Patil Medical College, Pune. A total of 600 individuals with confirmed TB either by sputum microscopy or Cartridge-based nucleic acid amplification test (CBNAAT) referred for WGS were included in the analysis. Patient identity was masked, and the data were barcoded before the analysis. A survey form was also sent to 42 healthcare providers to inquire about reasons for referral. Responses were received from 21 providers.

### DNA extraction

DNA extraction was carried out from decontaminated specimens enriched in Bactec MGIT 960 culture tubes (BD Instrument Systems, Sparks, MD, USA) using Qiagen DNeasy UltraClean Microbial Kit (Qiagen, Germany).

### WGS

The DNA libraries were prepared using Nextera system XT DNA Library Preparation kit (Illumina, San Diego, CA, USA) following the manufacturer’s instructions and were sequenced on the Illumina NovaSeq platform (Illumina)

### Bioinformatic analysis

Sequence alignment and mutation calling were done as reported earlier (11, 12). The pipeline involves a quality control step based on deduplication, Phred score of >30, and metagenomic binning for *Mycobacterium tuberculosis* complex. The binned reads which passed the QC metrics were then mapped to H37Rv using both Burrows-Wheeler aligner-maximum exact matches (13) and Genome Analysis Toolkit (14). A final variant calling list in SAMtools variable calling format was generated (15). The variants were annotated using a catalog curated from global catalogs (11). Lineages were identified based on the single-nucleotide polymorphism (SNP) classification scheme as indicated by Homolka et al (16). Phylogenetic analysis was done using the method elaborated by Zade et al. (11). Briefly, SNPs from individual samples were concatenated and subjected to multiple sequence alignment using multiple alignment using Fast Fourier Transform (https://www.ebi.ac.uk/Tools/msa/mafft/v7.310) (17). The alignment file was then used for phylogenetic reconstruction by the maximum-likelihood method in FastTree software (http://www.microbesonline.org/fasttree/v.2.1.11) (18). The phylogenetic trees were visualised using FigTree software (http://tree.bio.ed.ac.uk/software/figtree/v1.4.4) (19).

### Statistical analysis

Quantitative data were entered in MS Excel and later processed using SPSS version 27.0. We used *χ*^2^ test to compare proportions. Ages were represented as mean with standard deviation; Mann-Whitney *U* test was applied for comparison of ages between genders. Drug-resistance patterns are displayed graphically. We performed univariate and multivariate logistic regression analyses to identify risk factors associated with MDR-TB without variable selection unless otherwise stated in the results. For univariable and multivariable analyses, considering clinical significance, we grouped using the following categories: non-MDR-TB [drug susceptible (DS) and drug resistant (DR)] and for MDR-TB/MDR-TB+ [MDR-TB, pre-extensively drug-resistant tuberculosis (pre-XDR-TB) and XDR-TB].

## RESULTS

### Reasons for referral

Among the major reasons for patient referral for WGS, of 21 providers, 10 (private) mentioned multiple episodes of TB and non-response to the first-line anti-TB treatment, whereas the remaining 11 NTEP providers mentioned rifampicin resistance (RR) on CBNAAT/Xpert assay (3), family contacts of MDR-TB cases (3), non-response to first-line anti-TB treatment (3), and TB recurrence (2).

### Characteristics of the patients

WGS was successfully performed for all isolates from 600 patients. Of the 600 patients, 309 (52%) were men; the median age of the patients was 29 years (interquartile range 21–45). Statistically significant differences (*P* < 0.0001) were seen in the mean ages between males (39.6 yrs) and females (29.7 years). Samples from 30% patients did not show acid-fast bacilli on microscopy, and this was seen particularly for samples from women than men (34% vs 26%, *P* = 0.033) (Table 1). WGS identified a high proportion of pre-XDR-TB (50.83%), followed by MDR-TB (15.5%), with nearly equivalent proportions among men and women. More women than men were found to have drug-susceptible TB (23% vs 16%, *P* = 0.02757) (Table 1).

**TABLE 1:** Characteristics of study participants

Characteristics	Total, *n* (%)	Women, *n* (%)	Men, *n* (%)	*P* value
Cases studied	600	291 (48.5)	309 (51.5)	
Age group details^*a*^	598	290 (48.49)	308 (51.51)	
Age (years), mean ± SD	33.74 ± 15.7	29.57 ± 14.2	37.68 ± 16.08	<0.00001^*d*^
<14	24 (4.01)	17 (5.86)	7 (2.27)	0.02728
15–35	344 (57.52)	193 (66.55)	151 (49.02)	<0.00001^*d*^
36–56	166 (27.75)	62 (21.37)	104 (33.76)	0.0007230^*d*^
≥57	64 (10.70)	18 (6.20)	46 (14.93)	0.0005150^*d*^
Smear status^*b*^	551	264 (47.91)	287 (52.08)	
Not seen	166 (30.12)	91 (34.46)	75 (26.13)	0.03384
Acid fast bacilli seen (scanty)	23 (4.17)	14 (5.30)	9 (3.13)	
Acid fast bacilli seen (1+)	107 (19.41)	50 (18.93)	57 (19.86)	
Acid fast bacilli seen (2+)	107 (19.41)	43 (16.28)	64 (22.29)	
Acid fast bacilli seen (3+)	148 (26.86)	66 (25)	82 (28.57)	
Sample type^*b*^	551	264 (47.91)	287 (52.08)	
Pulmonary TB	509 (92.37)	241 (91.28)	268 (93.37)	
Extrapulmonary TB	42 (7.62)	23 (8.71)	19 (6.62)	
Drug profile	600	291 (48.5)	309 (51.5)	
DS	118 (19.66)	68 (23.36)	50 (16.18)	0.02757^*c*^
DR	40 (6.66)	17 (5.84)	23 (7.44)	
MDR-TB	93 (15.5)	40 (13.74)	53 (17.15)	
Pre-XDR-TB	305 (50.83)	143 (49.14)	162 (52.42)	
XDR- TB	44 (7.33)	23 (7.90)	21 (6.79)	

^*a*^ Results analyzed with data available for 598 of 600 participants.

^*b*^ Results analyzed with data available for 551 of 600 samples.

^*c*^ *P* < 0.05; *χ*^2^ tests, significant difference between women and men.

^*d*^ *P <* 0.01; *χ*^2^ test, highly significant difference between women and men.

### Drug resistance and lineages

**Fig 1:**
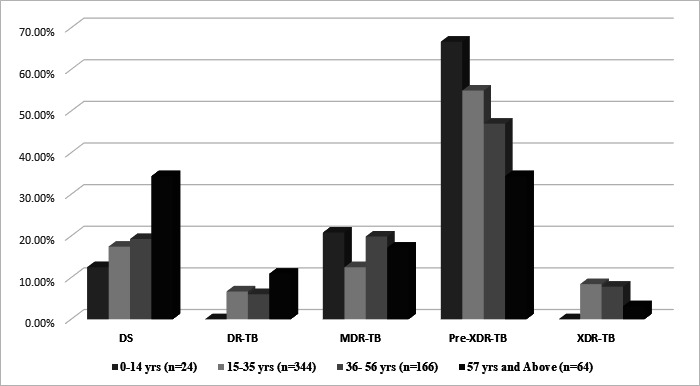
Drug -resistance status across age groups. Abbreviations: DR-TB, drug-resistant tuberculosis; DS, drug susceptible; MDR-TB, multidrug-resistant tuberculosis; pre-XDR-TB, pre-extensively drug-resistant tuberculosis; XDR-TB; extensively drug-resistant tuberculosis.

**Fig 2:**
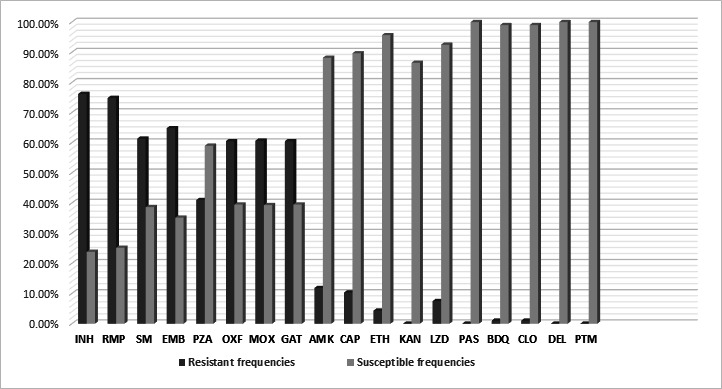
Drug-resistance frequency identified by WGS across the 18 antibiotics. Abbreviations: AMK, amikacin; BDQ, bedaquiline; CAP, capreomycin; CLO, clofazimine; DEL, delamanid; EMB, ethambutol; ETH, ethionamide; GAT, gatifloxacin; INH, isoniazid; KAN, kanamycin; LZD, linezolid; MOX, moxifloxacin; OXF, ofloxacin; PAS, para-amino salicylic acid; PTM; pretomanid; PZA, pyrazinamide; RMP, rifampicin; SM, streptomycin.

A high proportion of pre-XDR-TB was identified across different age groups: 55% in the age group from 15 to 35 years and 67% in the age group up to 14 years (Table 2; Fig. 1). A major proportion of isolates were resistant to the first-line anti-TB drugs, INH (76.16%), RMP (74.83%), ethambutol (EMB) (64.83%), and pyrazinamide (PZA) (41%). A high level of resistance to streptomycin (SM) (61.33%) was also observed, along with resistance toward fluoroquinolones (FQs) (60.5%). Resistance to LZD (45 of 600, 7.5%) and BDQ (6 of 600, 1%) was observed (Table 3; Fig. 2).

**TABLE 2:** DR categories across age groups

Age group (years)^*a*^	DS, *n* (%)	DR, *n* (%)	MDR, *n* (%)	Pre-XDR, *n* (%)	XDR, *n* (%)	*P* value
0–14 (*n* = 24)	3 (12.50)	0 (0)	5 (20.83)	16 (66.66)	0 (0)	0.008^*b*^
15–35 (*n* = 344)	60 (17.44)	23 (6.68)	43 (12.50)	189 (54.94)	29 (8.43)	
36–56 (*n* = 166)	32 (19.27)	10 (6.02)	33 (19.87)	78 (46.98)	13 (7.83)	
≥57 (*n* = 64)	22 (34.38)	7 (10.93)	11 (17.18)	22 (34.38)	2 (3.12)	

^*a*^ Results analyzed with data available for 598 of 600 samples.

^*b*^ *P* < 0.01, *χ*^2^ test.

**TABLE 3:** Frequency of resistance toward 18 drugs as identified by WGS and age

	INH (%)	RMP (%)	SM (%)	EMB (%)	PZA (%)	OXF (%)	MOX (%)	GAT (%)	AMK (%)	CAP (%)	ETH (%)	KAN (%)	LZD (%)	PAS (%)	BDQ (%)	CLO (%)	DEL (%)	PTM (%)
Resistance cases total	457 (76.16)	449 (74.83)	368 (61.33)	389 (64.83)	246 (41)	363 (60.5)	364 (60.66)	363 (60.5)	71 (11.83)	62 (10.33)	26 (4.33)	81 (13.5)	45 (7.5)	0 (0)	6 (1)	6 (1)	0 (0)	0 (0)
Resistance age-wise (years)^*a*^																		
0–14 (*n* = 24)	21 (87.50)	21 (87.50)	20 (83.33)	18 (75)	12 (50)	16 (66.67)	16 (66.67)	16 (66.67)	2 (8.33)	2 (8.33)	0 (0)	2 (8.33)	0 (0)	0 (0)	0 (0)	0 (0)	0 (0)	0 (0)
15–35 (*n* = 344)	268 (77.91)	266 (77.33)	216 (62.79)	235 (68.31)	136 (39.53)	226 (65.69)	227 (65.99)	226 (65.69)	41 (11.91)	38 (11.04)	16 (4.65)	54 (15.69)	28 (8.14)	0 (0)	4 (1.16)	5 (1.45)	0 (0)	0 (0)
36–56 (*n* = 166)	129 (77.71)	126 (75.90)	100 (60.24)	110 (66.27)	77 (46.39)	94 (56.62)	94 (56.62)	94 (56.62)	25 (15.06)	20 (12.05)	8 (4.82)	22 (13.25)	15 (9.04)	0(0)	2 (1.20)	1 (0.60)	0 (0)	0 (0)
57 and above (*n* = 64)	38 (59.38)	35 (54.69)	31 (48.44)	25 (39.06)	20 (31.25)	27 (42.19)	27 (42.19)	27 (42.19)	3 (4.69)	2 (3.13)	2 (3.13)	3 (4.69)	2 (3.13)	0(0)	0(0)	0(0)	0 (0)	0 (0)

^*a*^ Results analyzed with data available for 598 of 600 samples.

**Fig 3:**
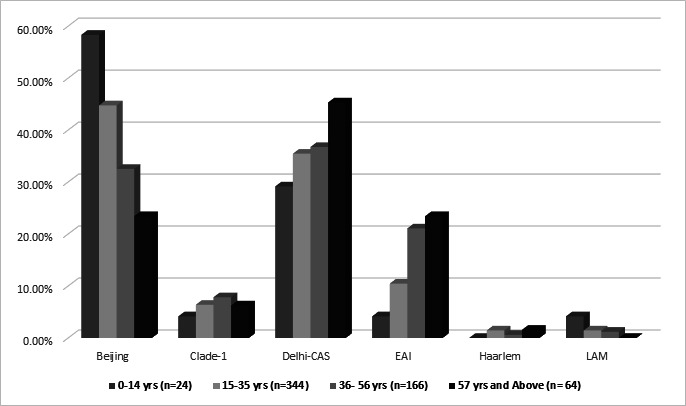
Distribution of lineages across age groups.

Samples from western India showed a high level of drug resistance to first-line anti-TB drugs like INH (79.31%), RMP (78.57%), EMB (67.24%), and also SM (66.01%) and the FQs [ofloxacin (61.33%), MOX (61.57%), and gatifloxacin (61.57%)]. Moreover, these also exhibited resistance to second-line injectable drugs (SLIDs), BDQ (1.23%), and clofazimine (1.47%). Drug resistance across different geographical regions is indicated in Table S1. The distribution of lineage across age groups is shown in Fig. 3.

We applied the classification system of Homolka et al. (16) for identification of lineages. The lineage informative SNP sets by this classification are largely compatible and unambiguously report the main lineages and sub-lineages when compared with other classification systems (Table S2).

**Fig 4:**
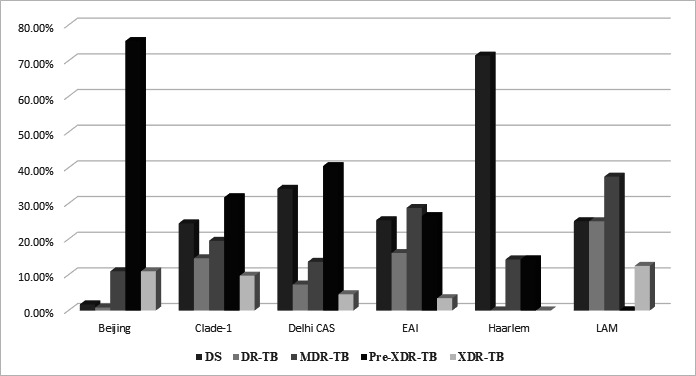
Frequency of drug-resistance categories across lineages in 600 cases.

The predominant lineage was the Beijing genotype (39.5%) followed by Delhi-Central Asian Strain (CAS) (36.66%) and East African Indian (EAI) (14.50%) (Table 4). Differences in lineage distribution between men and women were significant for Delhi-CAS (*P* = 0.024) and EAI (*P* = 0.032), but the distribution of all the lineages combined across the genders was not significant (*P* = 0.08). Significant differences were also seen in lineages across age groups (*P* = 0.009), with a higher proportion of Beijing lineage between 0 and 14 years (58%) and between 15 and 35 years (44.76%). Differences in drug resistance across lineages were also highly significant, with differences seen in Beijing lineage for DS, MDR, pre-extensively drug-resistant (pre-XDR) and XDR, and also in Delhi-CAS across the same categories. The pre-XDR-TB cases were mainly detected in isolates of the Beijing lineage (75.52%), followed by Delhi-CAS (40.45%). EAI lineage also exhibited significant differences with a lower proportion of pre-XDR and XDR isolates (Table 4; Fig. 4).

**TABLE 4:** Frequency of lineages across gender, age, and drug-resistance status^*c*^

Lineages	Beijing, *n* (%)	Clade 1, *n* (%)	Delhi-CAS, *n* (%)	EAI, *n* (%)	Haarlem, *n* (%)	LAM, *n* (%)
No. of cases	237 (39.5)	41 (6.83)	220 (36.66)	87 (14.5)	7 (1.16)	8 (1.33)
Females (*n* = 291)	116 (39.86)	15 (5.15)	120 (41.23)	33 (11.34)	3 (1.03)	4 (1.37)
Males (*n* = 309)	121 (39.15)	26 (8.41)	100 (32.36)	54 (17.47)	4 (1.29)	4 (1.29)
Sp lineage differences	*χ*^2^ = 0.0311, *P* = 0.860	*χ*^2^ = 2.501, *P* = 0.1137	***χ*^2^ = 5.082, *P* = 0.0241**	***χ*^2^ = 4.550, *P* = 0.032**	*χ*^2^ = 0.090, *P* = 0.763	*χ*^2^ = 0.007, *P* = 0.931
Combined	*χ*^2^ (5, *N* = 600) = 9.555, *P* = 0.088					
Age-wise (years), *n* (%)^*a*^						
0–14 (*n* = 24)	14 (58.33)	1 (4.16)	7 (29.16)	1 (4.16)	0 (0)	1 (4.16)
15–35 (*n* = 344)	154 (44.76)	22 (6.39)	122 (35.46)	36 (10.46)	5 (1.45)	5 (1.45)
36–56 (*n* = 166)	54 (32.53)	13 (7.83)	61 (36.74)	35 (21.08)	1 (0.60)	2 (1.20)
≥57 (*n* = 64)	15 (23.42)	4 (6.25)	29 (45.31)	15 (23.42)	1 (1.56)	0 (0)
Combined	***χ*^2^ (15, *N* = 598) = 30.656884, *P* = 0.0097**					
Drug resistance, *n* (%)^*b*^						
DS (*n* = 118)	4 (3.38)	10 (8.47)	75 (63.56)	22 (18.64)	5 (4.23)	2 (1.69)
DR (*n* = 40)	2 (5)	6 (15)	16 (40)	14 (35)	0 (0)	2 (5)
MDR (*n* = 93)	26 (27.96)	8 (8.6)	30 (32.26)	25 (26.89)	1 (1.07)	3 (3.23)
Pre-XDR (*n* = 305)	179 (58.69)	13 (4.26)	89 (29.18)	23 (7.54)	1 (0.33)	0 (0)
XDR (*n* = 44)	26 (59.09)	4 (9.09)	10 (22.72)	3 (6.82)	0 (0)	1 (2.27)
Lineage and resistance						
DS vs all	***P* < 0.00001**	*P* = 0.430	***P* < 0.00001**	*P* = 0.153	***P* = 0.0005**	*P* = 0.702
DS vs DR	*P* = 0.645	*P* = 0.237	*P* = 0.009	*P* = 0.055	**–**	*P* = 0.250
DS vs MDR	***P* < 0.00001**	*P* = 0.973	***P* < 0.00001**	*P* = 0.153	*P* = 0.1700	*P* = 0.467
DS vs PreXDR	***P* < 0.00001**	*P* = 0.086	***P* < 0.00001**	***P* = 0,001**	***P* = 0.002**	**–**
DS vs XDR	***P* < 0.00001**	*P* = 0.901	***P* < 0.00001**	***P* = 0.001**	–	*P* = 0.808
Combined	***χ*^2^ (20, *N* = 600) = 183.973, *P* < 0.00001**					

^*a*^ Results analyzed with data available for 598 of 600 samples.

^*b*^ Results analyzed with data available for 600 samples. *χ*^2^ analysis was done for lineage-specific differences between genders by determining the difference in the proportion of specific lineage gender-wise vs the remaining lineages; degree of freedom d(f) = 1, *n* = 600.

^*c*^ The bold faced values indicate statistical significance at P<0.01.

The univariate analysis indicated that younger age groups (<14, 15–35, and 36–56 years) and the Beijing strain were significant predictors of MDR-TB or MDR-TB+ (pre-XDR-TB and XDR-TB) (Table 5). However, multivariate analysis indicated that all young age groups, male gender, and Beijing genotype were significant independent predictors of MDR-TB or MDR-TB+ (Table 6). In other words, the multivariate analysis showed that children <14 years of age had nearly five times higher risk of developing or getting infected with MDR-TB/PreXDR or XDR-TB when compared with the age group of >57 years and young age groups (the age groups of 15–35 and 36–56 years have 2.5 times higher risk as compared to the age group of >57 years). Those infected with strains that belong to the Beijing lineage had nearly 44 times higher risk of MDR-TB/pre-XDR or XDR-TB than strains belonging to any other lineage. The classification table indicated that overall, 74.5% of cases (94.6% of MDR-TB, pre-XDR, and XDR) were correctly predicted through this model.

**TABLE 5:** Univariable logistic regression analysis: association with MDR-TB/MDR-TB+

Parameters (*n* = 598)	MDR-TB and MDR-TB+ (i.e., pre-XDR and XDR)	Non-MDR-TB	OR (95% CI^*b*^)	*P* value
Age group (years)	(*n* = 441)	(*n* = 157)		
<14	21	3	5.8 (1.5–21.4)	0.008^*a*^
15–35	261	83	2.6 (1.5–4.5)	0.001^*a*^
36–56	124	42	2.4 (1.3–4.4)	0.004^*a*^
>56	35	29	Ref.	
Gender				
Male	236	72	1.3 (0.9–1.9)	0.10
Female	205	85	Ref.	
Lineage				
Beijing	231	6	38.4 (7.7–191.7)	<0.0001^*a*^
Clade 1	24	16	1.5 (0.3–6.8)	0.602
Delhi-CAS	129	90	1.4 (0.3–5.8)	0.617
EAI	51	36	1.4 (0.3–6.04)	0.638
Haarlem	2	5	0.4 (0.04–3.4)	0.403
LAM	4	4	Ref.	

^*a*^ *P* < 0.01.

^*b*^ CI, confidence interval; OR, odds ratio.

**TABLE 6:** Multivariable logistic regression analysis: association with MDR-TB/MDR-TB+

Predictors of MDR/+	OR (95% CI)	*P* value
<14	4.6 (1.06–20.0)	0.042^*a*^
15–35	2.2 (1.1–4.2)	0.015^*a*^
36–56	2.5 (1.2–4.9)	0.004^*b*^
Male gender	1.6 (1.07–2.5)	0.022^*a*^
Beijing	44.7 (8.8–226.7)	<0.0001^*b*^
Clade 1	1.6 (0.3–7.6)	0.540
Delhi-CAS	1.7 (0.4–7.3)	0.439
EAI	1.6 (0.37–7.2)	0.5
Haarlem	0.45 (0.05–4.06)	0.457

^*a*^ *P* < 0.05.

^*b*^ *P* < 0.01.

### Mutations

**Fig 5:**
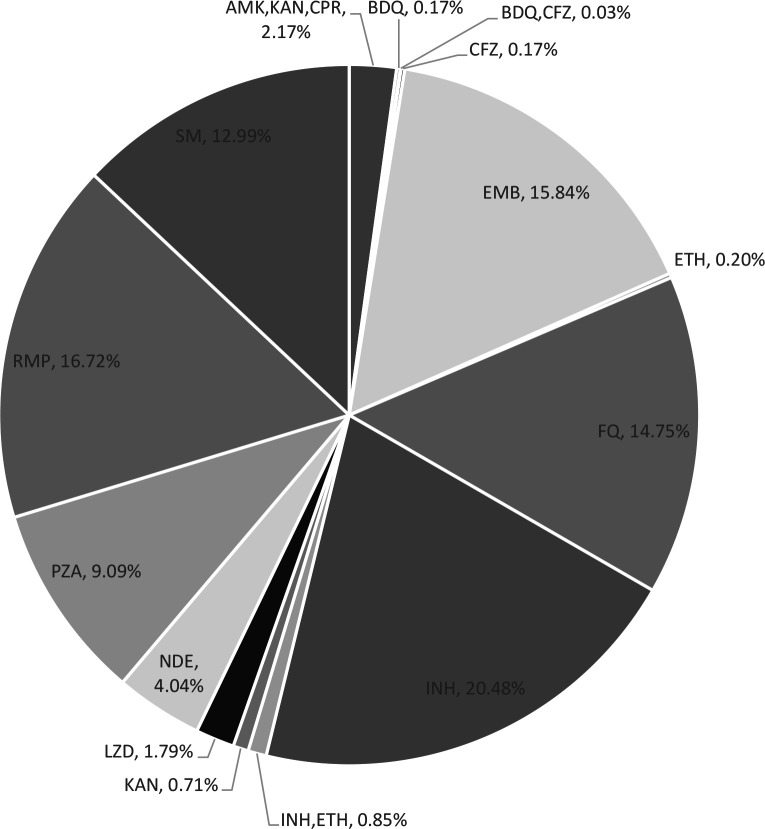
Frequency of mutations in 600 cases across the 18 antibiotics.

Maximum mutations were observed in regions known to encode resistance for RMP (16.72%), INH (20.48%), PZA (9.09%), SM (12.99%), and EMB (15.84%) (Fig. 5). Of the first-line drugs, rifampicin resistance was conferred predominantly by the Ser450Leu mutation (393 of 449, 87.5%), of which 74% (218 of 393) were in isolates of the Beijing lineage. Mutations outside the rifampicin resistance-determining region (RRDR) were seen in eight isolates (Ile491Phe and Ile170Val); disputed mutations in the *rpoB* were seen in 34 isolates (Table 7). The Ser315Thr katG mutation, prevalent at an average of 19% globally (20, 21) and associated with moderate- to- high-level resistance in isoniazid, was seen in 72.02% (435 of 600) of the isolates reported here. We also report the presence of both the n.C-15T in the fabG1 promoter (22) and the Ser315Thr katG mutations in 17% (100 of 600) of the isolates. Concurrent Ser315Thr katG mutation and ndh Arg268His mutation were seen in 1.5% of the isolates (Arg268His ndh mutation was observed in a total of 15 isolates). In total, 154 isolates exhibited a varied compound mutation profile, suggesting a high proportion of combination mutations in genes known to encode resistance to INH.

**TABLE 7:** Spectrum of common mutations lineage wise, disputed mutations, mutations outside RRDR

Drug	Substitution	Position	Gene name/gene symbol	Reference allele	Alternate allele	Beijing	Clade 1	Delhi-CAS	EAI	Haarlem	LAM	No. of cases
AMK, KAN and CPR	A1473246G	1473246	*rrs*	A	G	42 (67.74%)	4 (6.45%)	10 (16.13%)	5 (8.06%)	1 (1.61%)	–	62
BDQ	Ala63Pro (gca/Cca)	1461231	*atpE*	G	C	1 (33.33%)	–	1 (33.33%)	1 (33.33%)	–	–	3
BDQ	Asp15Gly (gac/gGc)	779033	*Rv0678*	A	G	–	–	–	1 (100%)	–	–	1
BDQ	Leu142Arg (ctg/cGg)	779414	*Rv0678*	T	G	1 (100%)	–	–	–	–	–	1
BDQ and CFZ	Ser53Leu (tcg/tTg)	779147	*Rv0678*	C	T	1 (100%)	–	–	–	–	–	1
CFZ	Ala102Val (gct/gTt)	779294	*Rv0678*	C	T	–	–	1 (100%)	–	–	–	1
CFZ	Arg156_ (cga/Tga)	779455	*Rv0678*	C	T	1 (100%)	–	–	–	–	–	1
CFZ	Gln51Arg (cag/cGg)	779141	*Rv0678*	A	G	1 (100%)	–	–	–	–	–	1
CFZ	Val20Phe (gtc/Ttc)	779047	*Rv0678*	G	T	2 (100%)	–	–	–	–	–	2
EMB	Gln497Arg (cag/cGg)	4248003	*embB*	A	G	59 (58.42%)	4 (3.96%)	29 (28.71%)	8 (7.92%)	1 (0.99%)	–	101
EMB	Met306Val (atg/Gtg)	4247429	*embB*	A	G	136 (62.67%)	4 (1.84%)	61 (28.11%)	15 6.91%)	–	1 0.46%)	217
ETH	Asp56Ala (gac/gCc)	4327307	*ethA*	T	G	–	–	1 (50%)	–	–	1 (50%)	2
ETH	Tyr84Asp (tac/Gac)	4327224	*ethA*	A	C	4 (100%)	–	–	–	–	–	4
FQ	Ala90Val (gcg/gTg)	7570	*gyrA*	C	T	48 (53.33%)	4 (4.44%)	26 (28.89%)	10 (11.11%)	1 (1.11%)	1 1.11%)	90
FQ	Asp94Gly (gac/gGc)	7582	*gyrA*	A	G	138 (59.74%)	6 (2.6%)	69 (29.87%)	18 (7.79%)	–	–	231
INH	C1673425T	1673425	*fabG1*	C	T	83 (68.03%)	2 (1.64%)	22 (18.03%)	14 (11.48%)	–	1 0.82%)	122
INH	Ser315Thr (agc/aCc)	2155168	*katG*	C	G	228 (52.41%)	29 (6.67%)	129 (29.66%)	43 (9.89%)	2 (0.46%)	4 0.92%)	435
INH and ETH	Arg268His (cgc/cAc)	2102240	*ndh*	C	T	1 (5.56%)	–	–	17 (94.44%)	–	–	18
KAN	C2715342T	2715342	*eis*	C	T	2 (22.22%)	–	5 (55.56%)	2 (22.22%)	-	-	9
KAN	G2715346A	2715346	*eis*	G	A	8 (72.73%)	–	3 (27.27%)		–	–	11
LZD	Cys154Arg (tgt/Cgt)	801268	*rplC*	T	C	20 (57.14%)	4 (11.43%)	9 (25.71%)	2 (5.71%)	–	–	35
PZA	Gly132Ala (ggt/gCt)	2288847	*pncA*	C	G	53 (72.6%)	1 (1.37%)	15 (20.55%)	4 (5.48%)	–	–	73
PZA	Leu27Pro (ctg/cCg)	2289162	*pncA*	A	G	41 (74.55%)	1 (1.82%)	12 (21.82%)	1 (1.82%)	–	–	55
RMP	Ser450Leu (tcg/tTg)	761155	*rpoB*	C	T	218 (55.47%)	21 (5.34%)	112 (28.5%)	36 (9.16%)	2 (0.51%)	4 1.02%)	393
SM	Lys43Arg (aag/aGg)	781687	*rpsL*	A	G	218 (64.5%)	13 (3.85%)	82 (24.26%)	23 (6.8%)	1 (0.3%)	1 (0.3%)	338
Disputed mutations (rpoB)												
RMP	Asp435Tyr (gac/Tac)	761109	*rpoB*	G	T	5(41.66%)	1 (8.33%)	4 (33.33%)	2 (16.66%)	0 (0%)	0 (0%)	12
RMP	His445Asn (cac/Aac)	761139	*rpoB*	C	A	0 (0%)	0 (0%)	2 (50%)	2 (50%)	0 (0%)	0 (0%)	4
RMP	His445Leu (cac/cTc)	761140	*rpoB*	A	T	1 (33.33%)	1 (33.33%)	1 (33.33%)	0 (0%)	0 (0%)	0 (0%)	3
RMP	Leu430Pro (ctg/cCg)	761095	*rpoB*	T	C	0 (0%)	1 (16.66%)	4 (66.66%)	1 (16.66%)	0 (0%)	0 (0%)	6
RMP	Leu452Pro (ctg/cCg)	761161	*rpoB*	T	C	3 (33.33%)	0 (0%)	1 (11.11%)	5 (55.55%)	0 (0%)	0 (0%)	9
Mutations outside RRDR												
RMP	Ile491Phe (atc/Ttc)	761277	*rpoB*	A	T	0 (0%)	0 (0%)	3 (60%)	2 (40%)	0 (0%)	0 (0%)	5
RMP	Val170Phe (gtc/Ttc)	760314	*rpoB*	G	T	1 (33.33%)	0 (0%)	0 (0%)	2 (66.66%)	0 (0%)	0 (0%)	3

Resistance to pyrazinamide was predominantly conferred by the Gly132Ala and Leu27Pro mutations in the *pncA* gene (73 and 55 isolates, respectively), of which both mutations were detected in four isolates. A combination of Gly132Ala with other mutations in the *pncA* gene (Thr47Ala, Phe94Leu, Asp12Glu, Gln10Pro, and *Rv2043c* intergenic c. 2289252T>C) was seen in seven isolates. Mutations known to confer resistance to EMB were seen in ~65% (389 of 600) of the isolates, with the highest frequency being at the 306 position in the *embB* gene. The *embB* Met306Val mutation was found in 217 of the 600 isolates followed by *embB* Gln497Arg mutation in 101 of the 600 isolates. Resistance to SM was conferred by mutations in 61% (368 of 600) of the isolates, with the Lys43Arg in *rpsl* gene seen in 338 of the 368 isolates (91.8%), of which 218 isolates were of the Beijing lineage (64.49%) as compared to 120 isolates in all other lineages combined. We report a lower frequency of *rrs* mutations conferring SM resistance (6.52%, 24 of 383) compared to a previous report (23). Similarly, we observed a lower frequency of *gidB* gene mutations (3 of 383) as compared to a previous study (24). Twenty- four isolates had both Asp94Gly and Ala90 Val together, and a total of 65 isolates had more than 1 mutation, with 11 isolates having mutations in both the both *gyrA* and *gyrase B* (*gyrB*) genes. From the isolates which had *gyrB* mutations, four had Arg446Cys; three had Asn499Thr; and one isolate each having Thr500Asn, Ala504Thr, Asn499Asp, and Thr500Pro mutations. Since mutations in *gyrB* typically are seen co-occurring with *gyrA* mutations, it becomes difficult to delineate their exact contribution to resistance and have been associated with lower sensitivity and specificity to FQ resistance (Table 7) (25).

Mutations in the *rrs* and *eis* genes are associated with resistance to the SLIDs [amikacin (AMK), KAN, and capreomycin (CAP)], though other genes have also been implicated, such as *whiB7*, *vapC21*, and *tllyA*, their effects still require explanation with larger data sets (26). AMK resistance was conferred in ~11% (71 of 600) of the isolates (1401A>G in the *rrs* gene, associated with cross resistance to KAN and CAP), of which 42 were associated with the Beijing lineage. Mutations in the *eis* gene were seen in 20 isolates, with 11 having the 2715346G>A (−14G>A) and 9 with the 2715342C>T (−10C>T) mutations. The *eis* gene mutations in the promoter regions (−10C>T and −14G>A) seen in 20 isolates (3.3%) are known to result in a low-level minimum inhibitory concentration (MIC) increase which can overlap with the WHO-based critical concentrations and often result in discordance with phenotypic assays.

However, low resistance to BDQ was conferred by the Ala63Pro mutation in the *atpE* gene in three isolates and by mutations in the *Rv0678* (known to confer cross resistance to CFZ) in seven isolates. LZD resistance, predominantly encoded by the T460C/Cys154Arg mutation in the *rlc gene* (27–29), was seen in 35 isolates with a higher frequency in the Beijing lineage (57.14%). We also report the mutation in the *rrl* gene known to be associated with a 8- to 50-fold increase in MIC (30) in 10 isolates in the current report.

**Fig 6:**
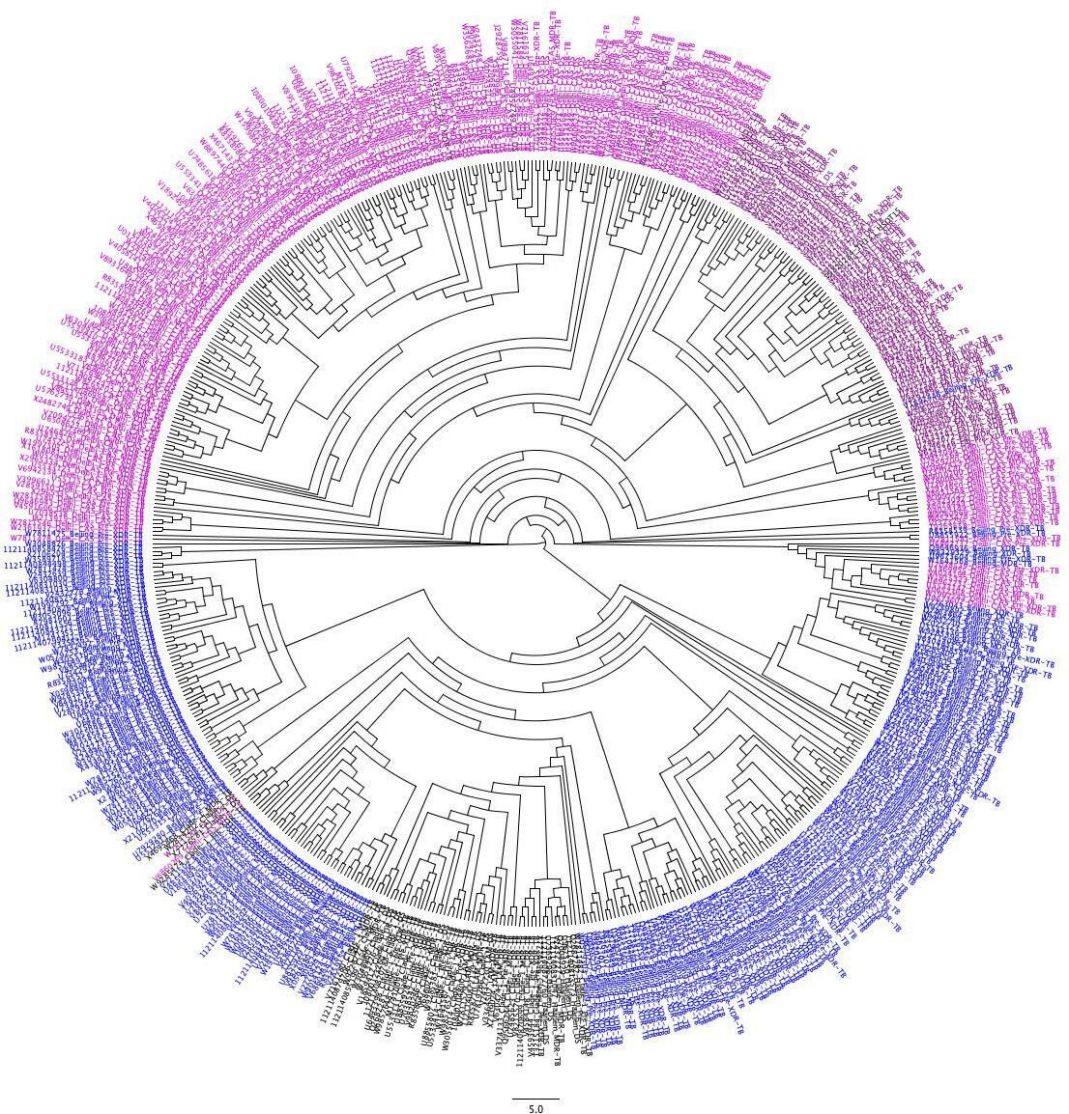
Phylogenetic analysis of 600 samples using concatenated SNP sequences. The lineages Delhi-CAS, EAI, Beijing, and Clade 1 are pink, purple, blue, and black, respectively. The phylogenetic reconstruction suggests that the clustering does not support the lineage-based clades, possibly due to the lineage-independent resistance overlapping SNPs other than phylogenetic markers.

SNP-based phylogenetic analysis suggests that, though the Beijing lineage is predominant in the study population, resistance in other lineages like EAI and Delhi CAS was also observed (Fig. 6; Fig. S1a through e).

## DISCUSSION

Here we report real-world data on the anti-TB drug-resistance profiles identified by WGS performed on *M.tb* isolated from confirmed TB patients from various geographical regions of India. There is an accumulating evidence that restricted access to drug-resistance diagnostics is fueling delays in appropriate care (31). In the absence of drug-susceptibility profile, patients are usually placed on therapy, which may contain a few drugs to which the *M.tb* strain is already resistant, and such therapy ultimately fails because of the risk of amplification of resistance (32). Such practices put communities at risk of exposure to increasingly resistant *M.tb*. Delays in diagnosis and an effective treatment of MDR-TB in the private sector are really a cause of concern (33). Despite a well-established national-level TB program that exists in India for more than three decades, the fact that the private sector plays a central role in TB management cannot be neglected. Interestingly, in the present study, we found that these patients were referred by the private practitioners for the reasons of non-response to the first-line treatment and/or multiple episodes of TB. Similarly, the NTEP providers reported RR on CBNAAT or recurrence as the main reasons for referral. The alarmingly high proportion of pre-XDR in these referrals and the existing cure rates for MDR and XDR-TB in India, which were at a level of 57% and 55%, respectively, in 2019 (5), are a testament to the fact that we need to address these issues on high priority. Studies by Atre et al. (33) in Pune and Atre et al. (34) in Mumbai reported that 72% and 62% of MDR-TB cases, respectively, belonged to the young and productive age group (15–35 years), hinting toward an ongoing transmission of drug-resistant strains in this region.

The data presented here also indicate similar findings where young age is associated with MDR/PreXDR/XDR TB. Moreover, in the present study, it was observed that even the middle-age group had a higher risk of being infected with highly resistant strains as compared to the age group of >57 years. These observations corroborate with the findings from a systematic review and meta-analysis (35) in Europe, which indicated that MDR-TB patients were younger than 65 years (odds ratio 2.53, 95% confidence interval (CI) 1.74 to 4.83). Furthermore, in the present study, we found that children <14 years had a higher risk of getting affected by the Beijing strain as compared to other strains. This observation was consistent with a study in Peru where Beijing strains were found more transmissible in children than are non-Beijing strains (36). This study further shows male gender as a significant predictor of MDR/Pre-XDR/XDR-TB, which is similar to the observations in the meta-analysis (35), but, in contrast to previous studies in Maharashtra state of India, shows that female gender was a significant predictor of MDR-TB (33, 34).

Despite the high burden of TB that is prevalent in India, representation of the genomic data is scarce. Garnering understanding of mutations especially for newer drugs and their resistance profiles in different geographic settings is critical, as is the need to constantly update databases. Here we report the presence of resistance-conferring mutations to RIF outside the RRDR region of the *rpoB* gene, which are not covered by rapid diagnostic tests (37) and are missed even by phenotypic DST (38, 39). This is a worrying trend and may lead to inaccurate diagnosis and underestimation of rifampicin resistance in the population (40). The mutation profile conferring resistance to INH showed high proportion of concurrent mutations and mutation conferring resistance to high dose of isoniazid (41, 42), further undermining the use of isoniazid in these cases. Double mutations of *inh*A and *kat*G reported here are also associated with increased risk at baseline for CAP resistance, XDR, and acquired FQ resistance as compared to mutations in *katG* only (43). We also detected multiple mutations conferring resistance to ethambutol were seen in 23 (Met306Val and Gln497Arg mutations in the *embB* gene) and 36 (Met306 in varied combinations) isolates, with a total of 72 isolates presenting >1 mutation.

Another concerning observation was the detection of mutations in the *pncA* gene in several isolates. Mutations in the *pncA* gene are shown to contribute to almost 72%–98% of resistance to PZA, along with known discordance between genotypic and phenotypic DST (44–46), due to the unreliable phenotypic DST of PZA (20, 47). The inclusion of PZA in several clinical trial regimens (Standardised Treatment Regimen of Anti-tuberculosis Drugs for Patients with Multidrug-resistant Tuberculosis [STREAM] and simplifying treatment for drug sensitive and-resistant tuberculosis [SIMPLICI TB]) and its combinatorial action with other drugs highlight the necessity of effectively determining resistance to it, especially since there is tripling of the mortality risk when individuals with resistance to PZA are put on a regimen with this component (48).

One of the most common drug resistances encountered in *M.tb* isolates worldwide is toward SM (49–51). SM is still used in several countries including India to complete the longer treatment regimen for MDR-TB and as a substitute for AMK. It is interesting to note that mutants Lys43Arg and Lys88Arg in *rpsl* exhibit high-level resistance, whereas mutants in *rrs* have low effect on MICs (52). Double mutations in *gyrA* and *gyrB* conferring resistance to FQ was observed in 11, which is in line with the 1%–3% range in previous reports (53–55).

FQ resistance is a major determinant of treatment failure in MDR patients. The referral patients for WGS in this study have shown a very high proportion of pre-XDR with FQ resistance causing mutations seen in 61% isolates. Global reports indicate roughly 60%–90% mutations being found in the quinolone determining resistance region codons 88–94 (53) of the *gyrase A (gyrA)* gene. In our study, 63% showed Asp94Gly mutation, and 25% had Ala90Val mutations. The presence of Asp94 mutations is sufficiently indicative of resistance; however, other mutations may result in MICs that may not be detected by the existing culture-based tests (56). We report a much higher FQ resistance (60%) as compared to the 21% FQ resistance reported in the national drug-resistance survey (57). Since our samples were predominantly from the individuals with suspected MDR cases, there could be a sample bias. A recent study in Mumbai showed a high proportion of FQ resistance among multidrug-resistant tuberculosis driven by dominant Lineage 2 *M.tb* clones (58), which includes the Beijing sub-lineage. Our study also reflects a high proportion of Beijing lineage and FQ resistance. A high level of FQ resistance is ubiquitous and is thus a cause of concern for India.

Another issue which is of concern is the presence of heteroresistance, which can cause treatment failures. Heteroresistance affects the resistance detection ability of rapid molecular tests, underestimating the true burden of FQ resistance. Here we reported that 60% (219 of 364) of the isolates with resistance-conferring mutations to FQs exhibited heteroresistance, with NGS reads ranging from 20% to 98% of the mutant alleles. In a previous study on five patients who were infected with ofloxacin-resistant TB and were treatment naïve with FQs, each carried mixed populations of resistant and sensitive bacilli (56). We also reported mutations in the *rrs* gene conferring resistance to KAN and CAP. Though these drugs are no longer used in treatment regimens, mutations reported in the *rrs* gene mutations are known to be significantly associated with XDR-TB (59).

Finally, a crucial finding in this report is the presence of mutations conferring resistance to BDQ and LZD. Both drugs are part of the shorter BPaL regimen for treating MDR-TB patients. The *atpE* Ala63Pro mutation reported here has been documented to cause a four- to eightfold increase in BDQ MICs with the DR-TB isolates (60, 61), which when present with mutations in the *Rv0678* gene may confer high-level BDQ resistance with an increased MIC (MIC of 2.0 and >8.0 mg/L in MGIT) (61).

The phylogenetic reconstruction using whole-genome SNPs of isolates reported here suggests that the accumulation of resistant mutations is an independent event. We could not establish an evolutionary transitional relationship between DS, DR, MDR, pre-XDR, and XDR strains in the study sample data set. Phylogenetic reconstruction from the DR, MDR, pre-XDR, and XDR samples in our data set showed lineage-based clustering, suggesting unbiased propensity of accumulating resistance imparting mutations among, Beijing, Delhi-CAS, EAI, and Clade 1. We speculate that the increasing incidences of MDR, pre-XDR, and XDR strains in different lineages could be due to the initiation of an antibiotic treatment regime based on an inconclusive diagnosis.

Implementation of WGS on clinical isolates of TB can have a positive impact on treatment changes and patient outcomes. Cox et al. (62), in a study of TB patients in South Africa, indicated that 46% (385 of 834) may have benefited from reduced drug dosage or removing ineffective drugs; a further 22% (187 of 834) may have benefited from effective adjusted regimens; and 35% (153) of the 440 patients eligible for longer regimen could be prescribed an effective shorter regimen. The impact on the outcome on accurate therapy guided by TB-WGS was also observed by Zürcher et al. (63), wherein from seven TB high-burden countries it was reported that underdiagnosis of drug resistance resulted in inappropriate treatment and higher mortality with an adjusted odds ratio of 4.92 (95% CI 2.47–9.78) among undertreated patients, compared with appropriately treated patients.

In that context, it will be important to study what strategies have been adapted by the clinicians after receiving the WGS results and how those impacted further treatment outcomes. Also studying the utility and cost-effectiveness of implementing WGS in an Indian setting would be a key step. In our opinion, the NTEP should take cognizance of these findings and initiate new studies to generate evidence for scale-up of WGS in near future.

### Conclusion

In conclusion, there are relatively few studies providing information of the mutation spectrum seen in *M.tb* from India, a country which contributes to almost one-third of TB cases. Though this study has limitations with respect to sampling bias, availability of phenotypic data, and clinical outcomes, to the best of our knowledge, this is one of the largest report to date which adds a significant body of information on the drug-resistant mutations from clinical referral samples in India and brings to attention the issue of emerging resistance to newer drugs such as bedaquiline. It underscores the need for training both care providers for rational use of anti-TB drugs and the patients.

## SUPPLEMENTAL MATERIAL

Fig. S1a spectrum.02770-23-s0001.tif Phylogenetic analysis of DS samples using concatenated SNP sequences.

Fig. S1b spectrum.02770-23-s0002.tif Phylogenetic analysis of DR samples using concatenated SNP sequences.

Fig. S1c spectrum.02770-23-s0003.tif Phylogenetic analysis of MDR samples using concatenated SNP sequences.

Fig. S1d spectrum.02770-23-s0004.tif Phylogenetic analysis of pre-XDR samples using concatenated SNP sequences.

Fig. S1e spectrum.02770-23-s0005.tif Phylogenetic analysis of XDR samples using concatenated SNP sequences.

Legends spectrum.02770-23-s0006.docx Legends for the supplemental figures.

Supplemental data spectrum.02770-23-s0007.xlsx Comparison of lineages by Homolka et al. with other classification system.

Table S1 spectrum.02770-23-s0008.docx Frequency of resistance towards 18 drugs as identified by WGS and region.

ASM does not own the copyrights to Supplemental Material that may be linked to, or accessed through, an article. The authors have granted ASM a non-exclusive, world-wide license to publish the Supplemental Material files. Please contact the corresponding author directly for reuse.
