# Supplementary figures and images for: Whole-genome sequencing of clinical isolates from tuberculosis patients in India: real-world data indicates a high proportion of pre-XDR cases
Source: Microbiol Spectr. 2024 Apr 10;12(5):e02770-23. doi: 10.1128/spectrum.02770-23 (PMC11064594; doi:10.1128/spectrum.02770-23)

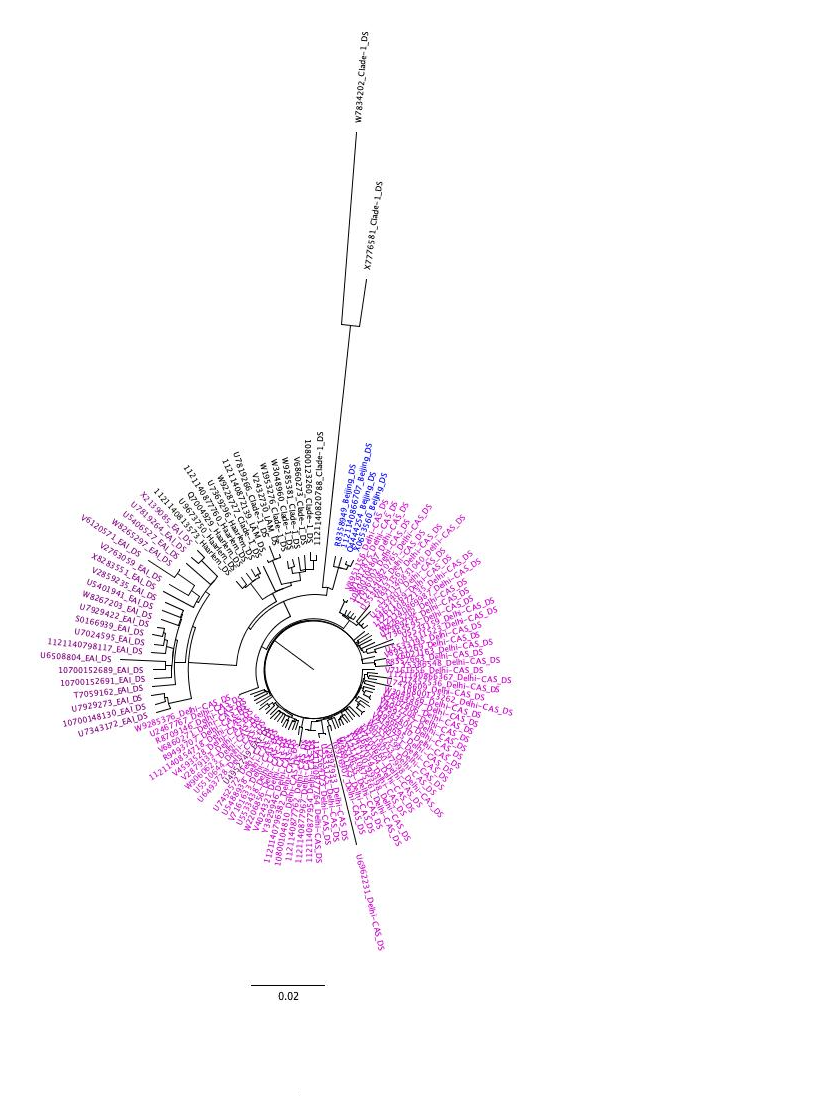

Supplement: Fig. S1a — Phylogenetic analysis of DS samples using concatenated SNP sequences. [file spectrum.02770-23-s0001.tif]

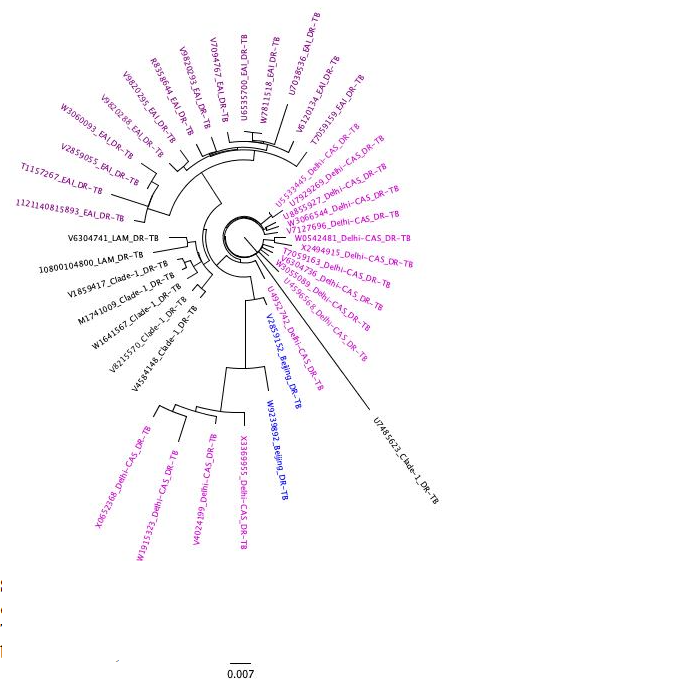

Supplement: Fig. S1b — Phylogenetic analysis of DR samples using concatenated SNP sequences. [file spectrum.02770-23-s0002.tif]

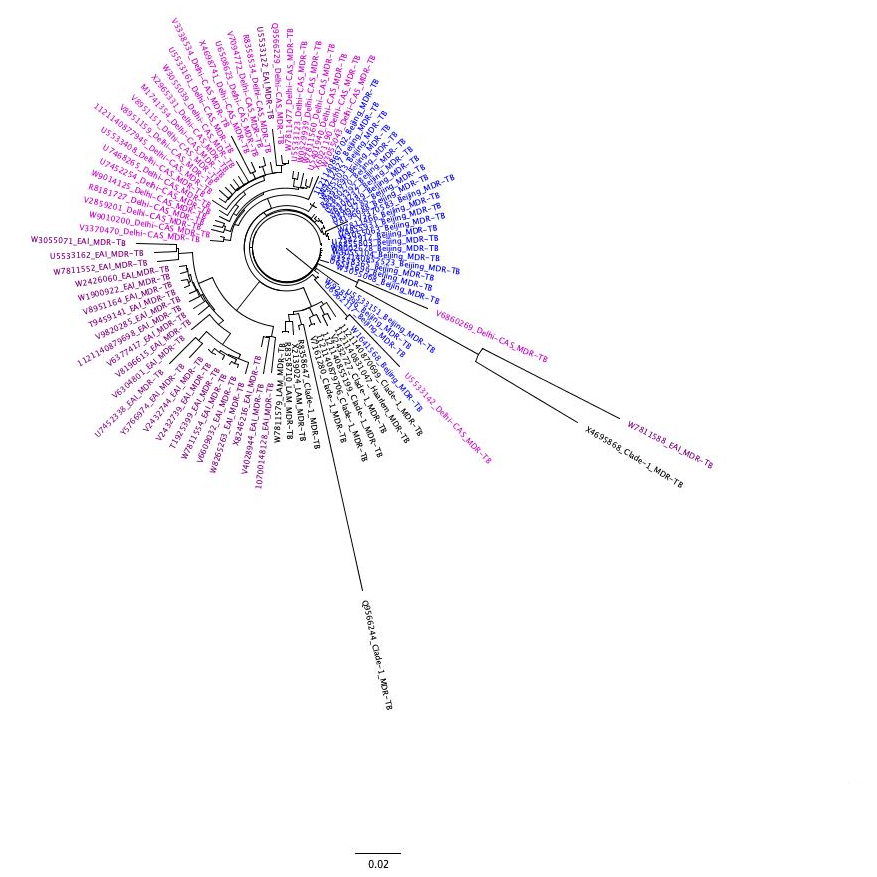

Supplement: Fig. S1c — Phylogenetic analysis of MDR samples using concatenated SNP sequences. [file spectrum.02770-23-s0003.tif]

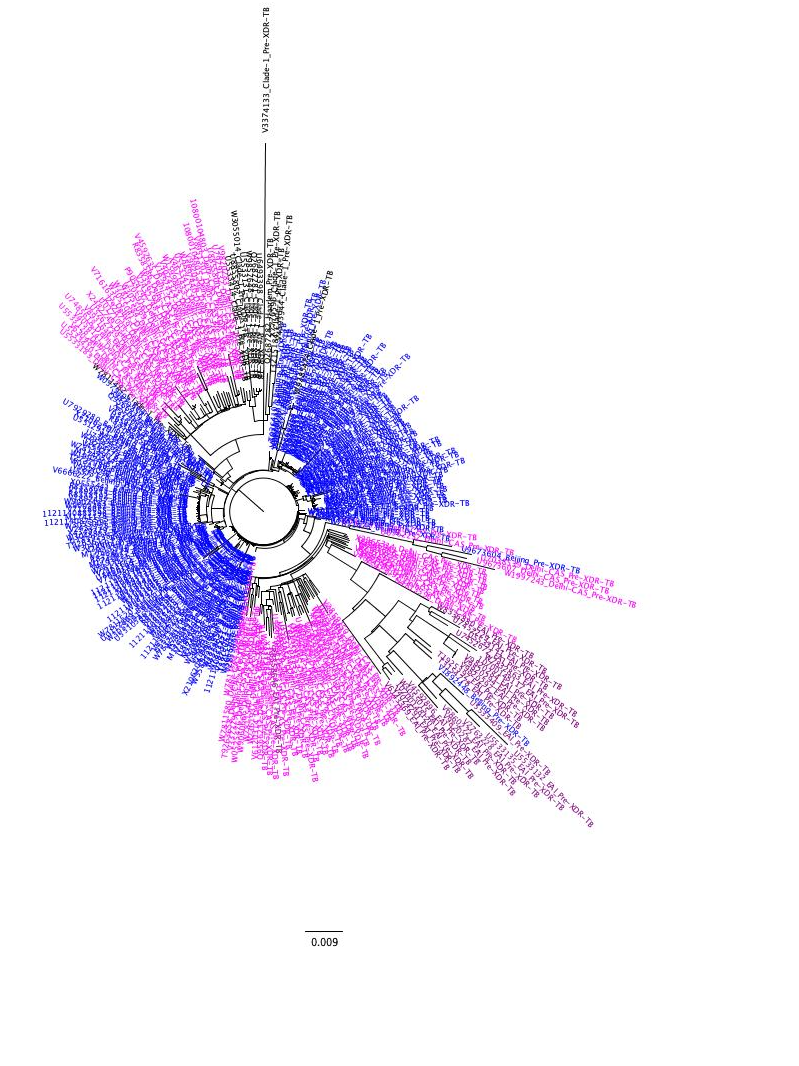

Supplement: Fig. S1d — Phylogenetic analysis of pre-XDR samples using concatenated SNP sequences. [file spectrum.02770-23-s0004.tif]

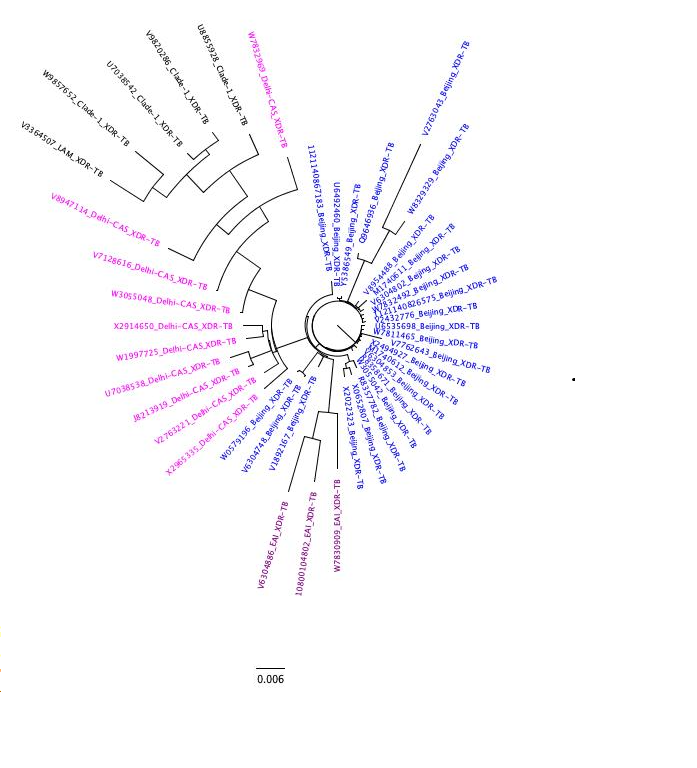

Supplement: Fig. S1e — Phylogenetic analysis of XDR samples using concatenated SNP sequences. [file spectrum.02770-23-s0005.tif]
