# Supplementary material for: Whole-genome sequencing of clinical isolates from tuberculosis patients in India: real-world data indicates a high proportion of pre-XDR cases
Source: Microbiol Spectr. 2024 Apr 10;12(5):e02770-23. doi: 10.1128/spectrum.02770-23 (PMC11064594; doi:10.1128/spectrum.02770-23)
Supplement: Legends — for the supplemental figures. [file spectrum.02770-23-s0006.docx]

**Supplementary Figures Legends:**

**Supplementary Figure 1a: Phylogenetic analysis of DS samples using concatenated SNP sequences.**

Abbreviations used: DS; drug sensitive. The lineages Delhi- CAS, EAI, Beijing, and Clade I are coloured pink, purple, blue and black respectively.

**Supplementary Figure 1b: Phylogenetic analysis of DR samples using concatenated SNP sequences.**

Abbreviations used: DR; drug resistant. The lineages Delhi- CAS, EAI, Beijing, and Clade I are coloured pink, purple, blue and black respectively.

**Supplementary Figure 1c: Phylogenetic analysis of MDR samples using concatenated SNP sequences.**

Abbreviations used: MDR; multi drug resistant. The lineages Delhi- CAS, EAI, Beijing, and Clade I are coloured pink, purple, blue and black respectively.

**Supplementary Figure 1d: Phylogenetic analysis of pre-XDR samples using concatenated SNP sequences.**

Abbreviations used: pre-XDR; pre- extensively drug resistant. The lineages Delhi- CAS, EAI, Beijing, and Clade I are coloured pink, purple, blue and black respectively.

**Supplementary Figure 1e: Phylogenetic analysis of XDR samples using concatenated SNP sequences.**

The lineages Delhi- CAS, EAI, Beijing, and Clade I are coloured pink, purple, blue and black respectively.
