# Supplementary material for: Whole-genome sequencing of clinical isolates from tuberculosis patients in India: real-world data indicates a high proportion of pre-XDR cases
Source: Microbiol Spectr. 2024 Apr 10;12(5):e02770-23. doi: 10.1128/spectrum.02770-23 (PMC11064594; doi:10.1128/spectrum.02770-23)
Supplement: Table S1 — Frequency of resistance towards 18 drugs as identified by WGS and region. [file spectrum.02770-23-s0008.docx]

**Supplementary Table 1: Frequency of Resistance towards 18 drugs as identified by WGS and Region**

| **Resistance region-wise** **^#^** | **INH**  **(%)** | **RMP**  **(%)** | **SM**  **(%)** | **EMB**  **(%)** | **PZA**  **(%)** | **OXF**  **(%)** | **MOX (%)** | **GAT**  **(%)** | **AMK**  **(%)** | **CAP (%)** | **ETH**  **(%)** | **KAN**  **(%)** | **LZD**  **(%)** | **PAS (%)** | **BDQ (%)** | **CLO**  **(%)** | **DEL**  **(%)** | **PTM**  **(%)** |
| --- | --- | --- | --- | --- | --- | --- | --- | --- | --- | --- | --- | --- | --- | --- | --- | --- | --- | --- |
| Central India (n=6) | 3  (50%) | 3  (50%) | 2 (33.33%) | 3  (50%) | 3  (50%) | 2 (33.33%) | 2 (33.33%) | 2 (33.33%) | 0  (0%) | 0  (0%) | 0  (0%) | 0  (0%) | 0  (0%) | 0  (0%) | 0  (0%) | 0  (0%) | 0  (0%) | 0  (0%) |
| Eastern India (n=25) | 18  (72%) | 17  (68%) | 12  (48%) | 17  (68%) | 9  (36%) | 16  (64%) | 16  (64%) | 16  (64%) | 3  (12%) | 2  (8%) | 1  (4%) | 4  (16%) | 3  (12%) | 0  (0%) | 1  (4%) | 0  (0%) | 0  (0%) | 0  (0%) |
| Northern India (n=96) | 71 (73.95%) | 67 (69.79%) | 58 (60.42) | 60 (62.5%) | 37 (38.54%) | 62 (64.58%) | 62 (64.58%) | 61 (63.54%) | 11 (11.45%) | 7 (7.29%) | 3 (3.12%) | 9 (9.37%) | 5 (5.20%) | 0  (0%) | 0  (0%) | 0  (0%) | 0  (0%) | 0  (0%) |
| Southern India (n=57) | 37 (64.91%) | 38 (66.66%) | 23 (40.35%) | 27 (47.36%) | 15 (26.31%) | 28 (49.12%) | 28 (49.12%) | 28 (49.12%) | 1 (1.75%) | 1 (1.75%) | 0 (0%) | 3 (5.26%) | 2 (3.50%) | 0  (0%) | 0  (0%) | 0  (0%) | 0  (0%) | 0  (0%) |
| Western India (n=406) | 322 (79.31%) | 319 (78.57%) | 268 (66.01%) | 273 (67.24%) | 180 (44.33%) | 249 (61.33%) | 250 (61.57%) | 250 (61.57%) | 53 (13.05%) | 50 (13.05%) | 22 (5.41%) | 62 (15.27%) | 34 (8.37%) | 0  (0%) | 5 (1.23%) | 6 (1.47%) | 0  (0%) | 0  (0%) |

*# Results analyzed with data available for 550 /600 samples*
